# Supplementary figures and images for: Comparative analysis of the complete plastid genomes of Mangifera species and gene transfer between plastid and mitochondrial genomes
Source: PeerJ. 2021 Feb 10;9:e10774. doi: 10.7717/peerj.10774 (PMC7881718; doi:10.7717/peerj.10774)

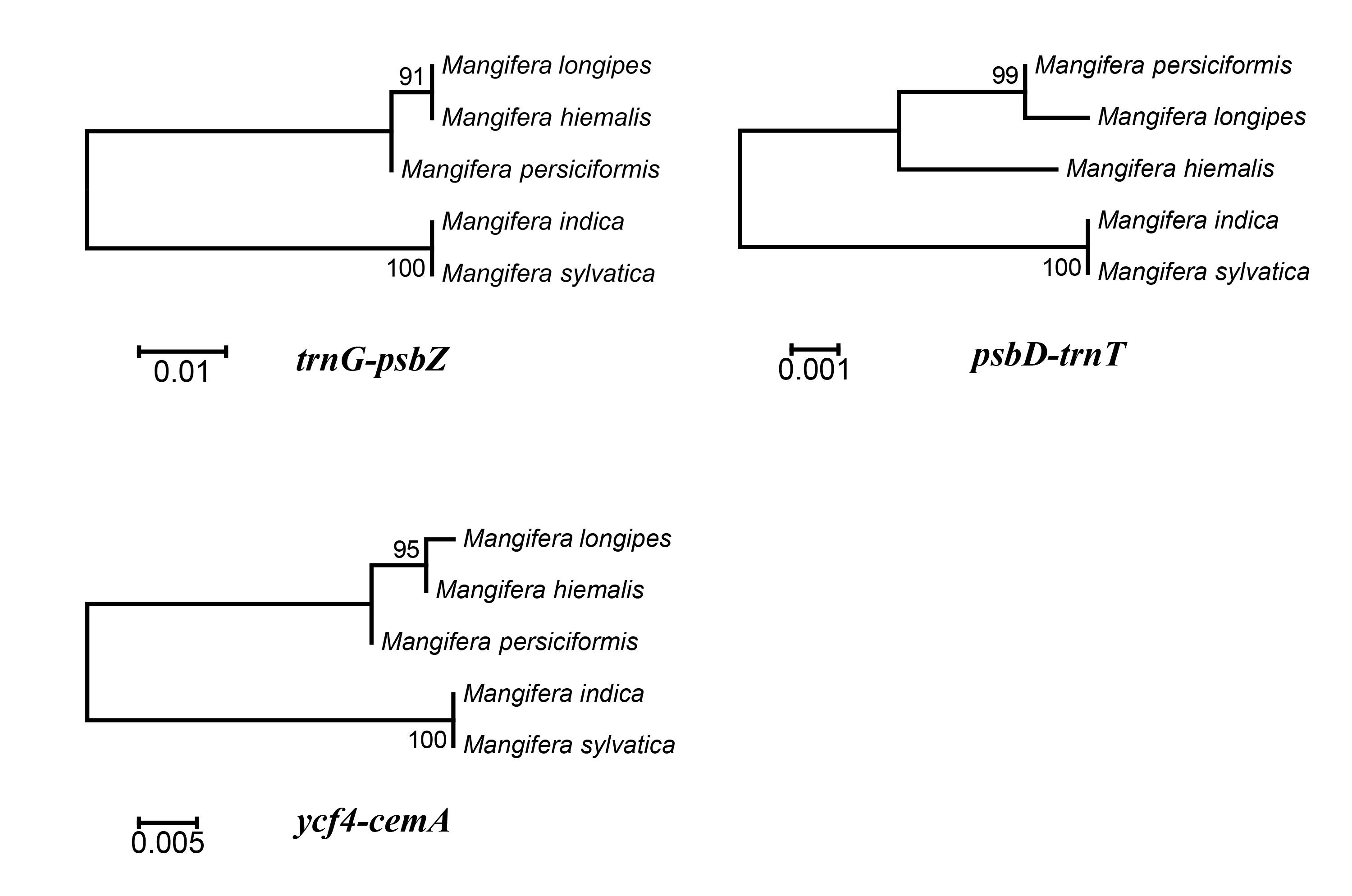

Supplement: Supplemental Information 9 [file peerj-09-10774-s009.png]

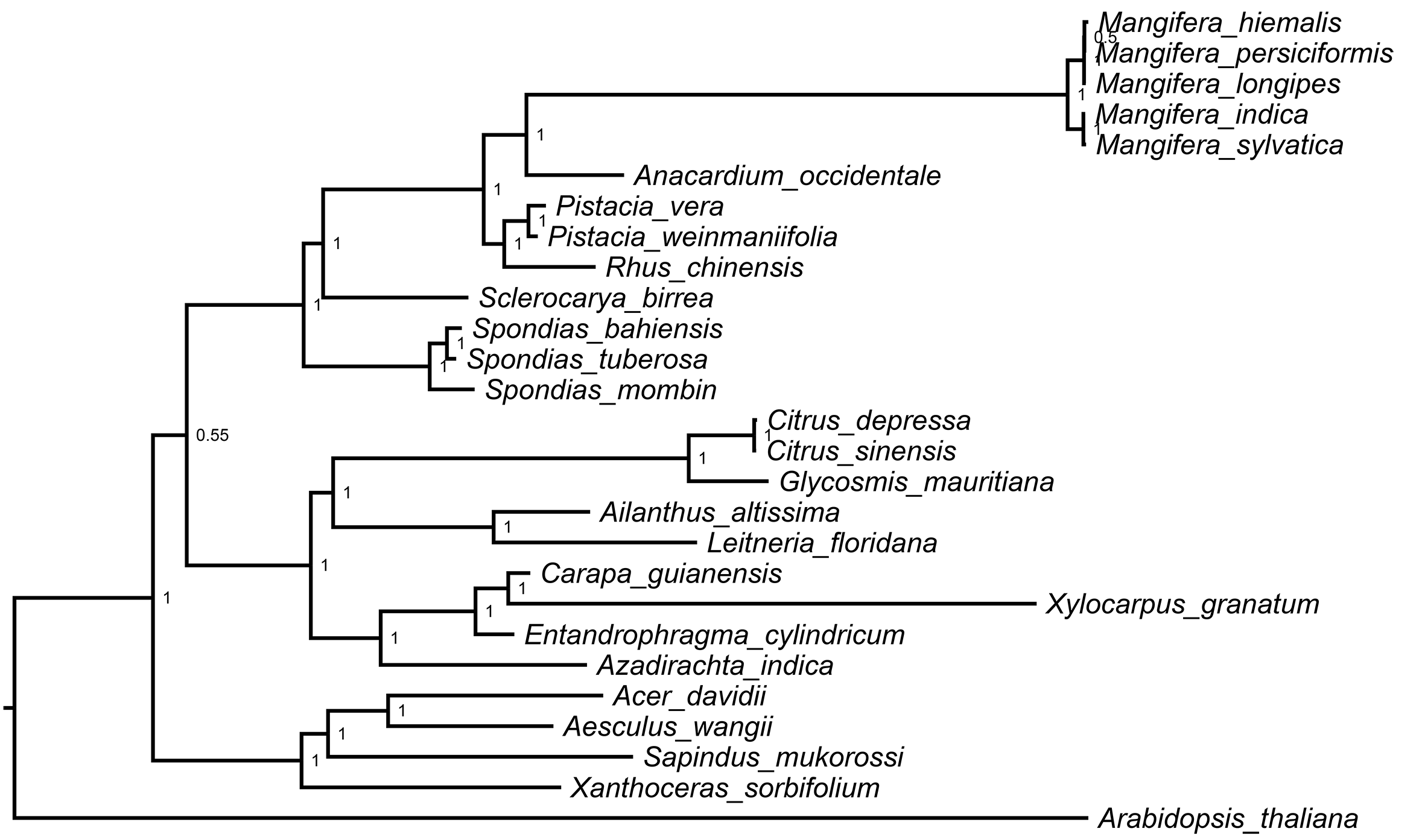

Supplement: Supplemental Information 10 [file peerj-09-10774-s010.png]

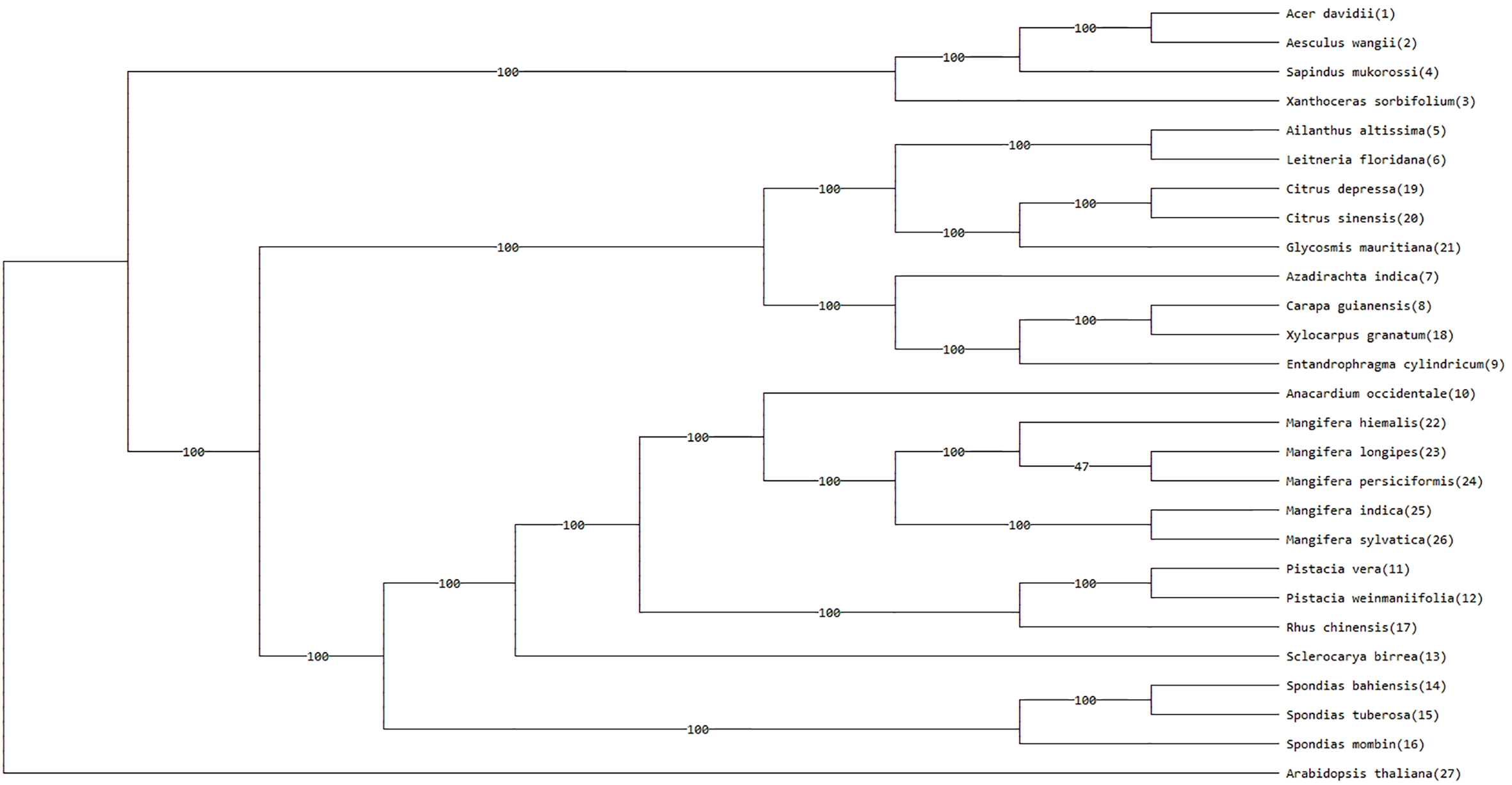

Supplement: Supplemental Information 11 [file peerj-09-10774-s011.png]

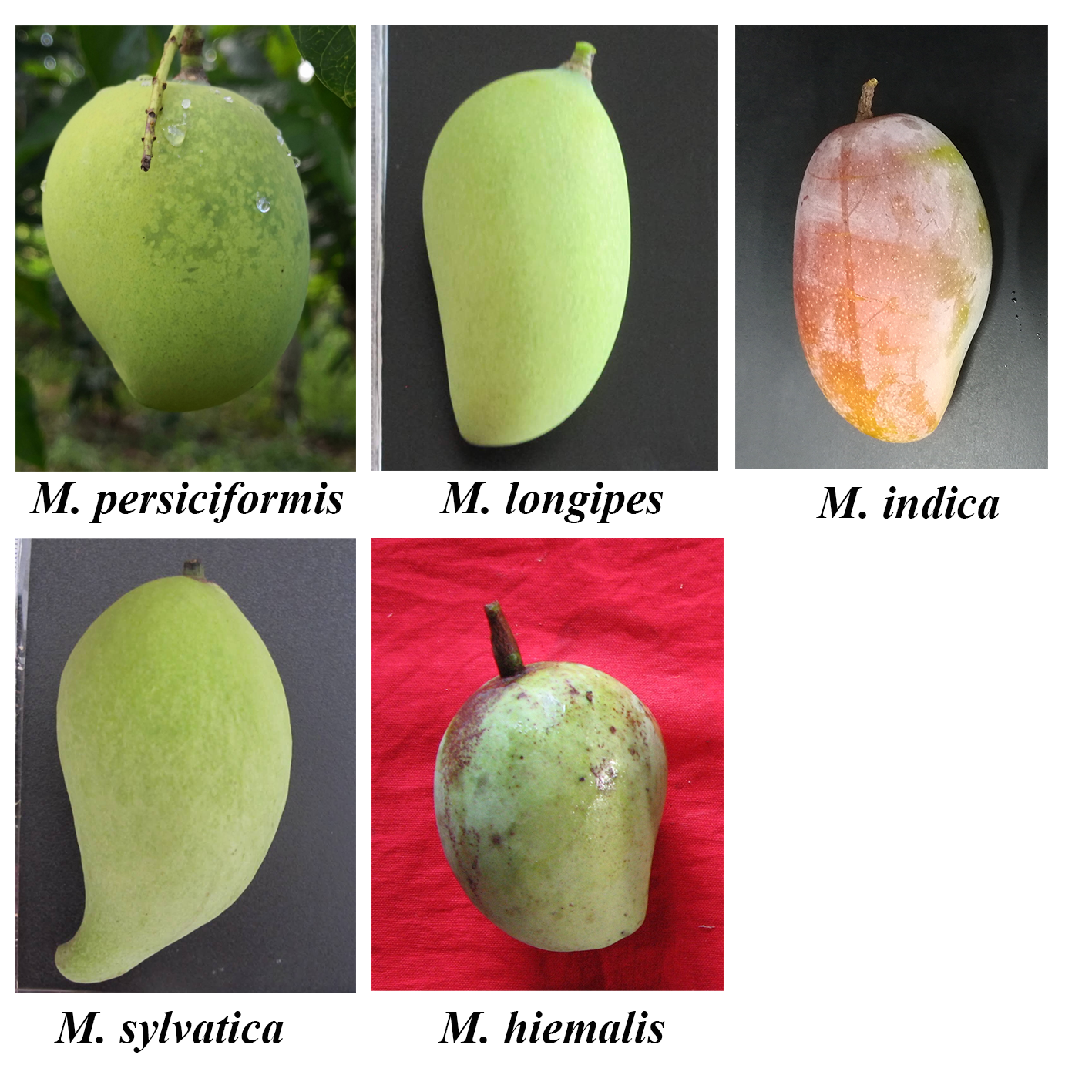

Supplement: Supplemental Information 12 [file peerj-09-10774-s012.png]
